# MicroRNA-610 inhibits tumor growth of melanoma by targeting LRP6

**DOI:** 10.18632/oncotarget.22125

**Published:** 2017-10-26

**Authors:** Guangjing Zhang, Dongfang Ai, Xiufang Yang, Shanshan Ji, Zhengxiang Wang, Shijun Feng

**Affiliations:** ^1^ Department of Dermatology, Cangzhou Central Hospital, Cangzhou, Hebei 061001, P.R. China

**Keywords:** microRNA-610, melanoma, LRP6, proliferation, apoptosis

## Abstract

Accumulating evidence showed that aberrant miRNAs expression was involved in initiation and progression of melanoma. However, the investigation of different miRNAs in melanoma remain attractive. In this research, we demonstrated that miR-610 expression was decreased in melanoma tissues and cell lines. The clinical data showed that the reduced miR-610 expression was obviously associated with adverse prognostic characteristics. Furthermore, our results suggested that miR-610 had a function of prognostic indicator for 5-year predicted-survival of melanoma patients. The ectopic overexpression of miR-610 suppressed cell proliferation, cell cycle progression and promoted apoptosis while miR-610 knockdown reversed the effect *in vitro* and *in vivo*. Additionally, miR-610 could modulate LRP6 by directly interacting to its 3’-UTR. In clinical samples of melanoma, miR-610 inversely correlated with LRP6. The biological function of miR-610 on melanoma cells was abrogated by alternation of LRP6 expression. In summary, our research indexed that miR-610 had a function of tumor suppressor in regulating the proliferation, cell cycle and apoptosis of melanoma via targeting LRP6. Hence, it may represent a novel potential therapeutic target and prognostic marker for melanoma.

## INTRODUCTION

Melanoma is a highly aggressive and malignant tumor with an increasing incidence rate in recent years [1, 2]. Despite the advancement in surgery resection, radiotherapy, chemotherapy and pathogenesis of melanoma, the long-term survival and prognosis of melanoma remains extremely low because of its recurrence and metastasis [3, 4]. Its therapeutic outcome relies on early diagnosis and effective treatment, including surgical resection [5, 6]. Using tissue-engineered melanoma models, the melanoma and tissue surrounding melanoma can be detected by Raman spectroscopy [7]. Therefore, it is important to find novel efficient biomarkers to develop therapeutic strategies and prognosis.

MicroRNAs (miRNAs) can post-transcriptionally regulate target gene expression by binding complementary sequence with 3’ untranslated region (3’-UTR) to induce the degradation of mRNA or inhibit the translation of mRNA [8-10]. Mounting evidence confirmed that dysregulated miRNAs are involved the progression of cancers [11, 12], including melanoma [13, 14], and regulate cellular biological progress such as proliferation, cell cycle, apoptosis and metastasis. HAcat cells proliferation and migration can be inhabited by miR-26a through regulating PTEN expression [15]. Recently, miR-610 has been recognized as cancer-related miRNA. It has been reported that miR-610 has a suppression on the expression of vasodilator-stimulated phosphoprotein resulting in inhibition of migration and invasion of gastric cancer cells [16]. Downregulation of miR-610 promotes proliferation and tumorigenicity and activates Wnt/β-catenin signaling in human hepatocellular carcinoma through directly suppressing lipoprotein receptor-related protein 6 (LRP6) and transducin β–like protein 1 (TBL1X) [17]. Moreover, by targeting Twist1 expression, miR-610 also could restrain proliferation, cycle, invasion, EMT program and drug-sensitivity of osteosarcoma cells [18]. In colorectal cancer, cell proliferation and invasion are also inhibited through the method of repressing hepatoma-derived growth factor by miR-610 [19]. Whereas, the potential mechanisms involved in the development of melanoma is not completely clear, and the importance of miR-610 in clinic treatment remain to be investigated.

Here, we aimed to explore the expression and biological function of miR-610 in the melanoma in the research. And our results confirmed that the expression of miR-610 was down-regulated in the melanoma cell lines and tissues. Overexpression of miR-610 suppressed, while miR-610 knockdown promoted cell proliferation, cell cycle and apoptosis resistance *in vitro* and *in vivo*.. Furthermore, it was recognized that LRP6 is a direct target of miR-610. Thus, we demonstrated that miR-610 play a crucial part in melanoma progression and represent a latent biomarker for melanoma diagnosis and therapy.

## RESULTS

### Down-regulation of miR-610 in melanoma cell lines and tissues

**Figure 1:**
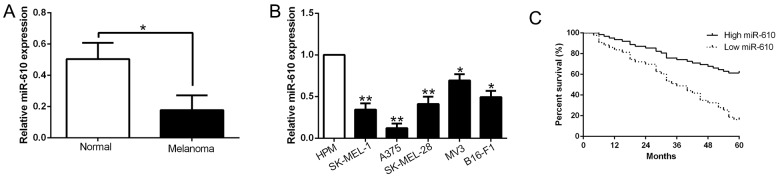
The decrease of miR-610 in melanoma cell lines and tissues, and the significance of decline in prognosis prediction **(A)** qRT-PCR was employed for assaying related miR-610 levels in melanoma tissues and matched adjacent non-tumor tissues. **(B)** Comparing with the HPM cells, miR-610 expression was signally enhanced in five melanoma cell lines. The internal control was U6 snRNA. **(C)** Using Kaplan-Meier analyzed the prognostic value of miR-610 for melanoma patients. **P< 0.01, *P < 0.05.

To explore the potential expression level of miR-610, we measured the miR-610 expression pattern in melanoma cell lines and tissues. And Figure 1 shown the result. Comparing with adjacent non-tumor tissues, miR-610 expression in melanoma tissues was evidently down-regulated (P<0.05, Figure 1A). Comparing with human primary melanocytes (HPM), decrease of miR-610 expression was obviously observed in melanoma cell lines (P<0.05, Figure 1B). The data manifested that the reductive miR-610 participates the life action of melanoma.

### Clinical significance of miR-610 in melanoma

According the mid-value of miR-610 expression, we divided the patients into two groups to inquired the relationship between clinicopathological features and prognosis. As the results shown in Table 1, advanced tumor stage (P=0.009) and thickness (P=0.002) of melanoma were obviously related to the expression of miR-610. Furthermore, high expression patients had a meliorative survival than those with low expression (P<0.05, Figure 1C). These results indicated that miR-610 could do duty for a potential prognostic biomarker in melanoma patients.

**Table 1:** Clinical correlation of miR-610 expression in melanoma (n = 105)

Clinical parameters	Cases (n)	Expression level		*P* value (^*^p<0.05)
		miR-610^high^ (n=52)	miR-610^low^ (n=53)	
Age (years)				
< 65 years	43	20	23	0.607
≥65 years	62	32	30	
Gender				
Male	60	31	29	0.612
Female	45	21	24	
Tumor thickness				0.002^*^
<1	47	31	16	
≥1	58	21	37	
Ulceration				0.750
negative	90	44	46	
positive	15	8	7	
LNM				
negative	55	31	24	0.141
positive	50	21	29	
Tumor stage				0.009^*^
I/II	32	22	10	
III	73	30	43	

LNM: lymph node metastasis.

### The biological effects of miR-610 in melanoma progression

**Figure 2:**
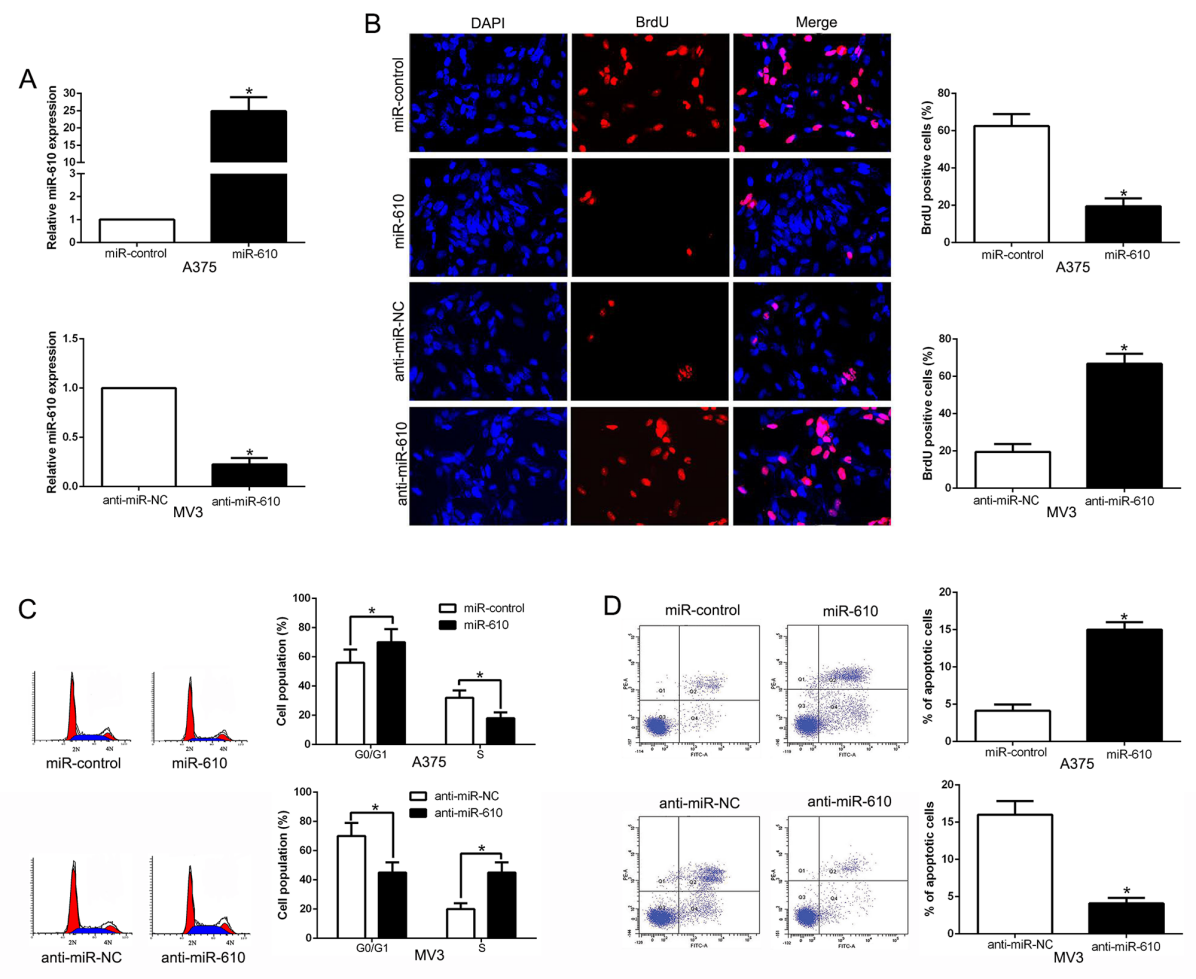
The biological effects of miR-610 expression **(A)** The expressions of miR-610 transduced with corresponding miRNA vectors in A375 cells and MV3 cells were measured by qRT-PCR. The effects of miR-610 overexpression in A375 cells: **(B)** proliferation inhibition, **(C)** cycle progression inhibition and **(D)** apoptosis promotion. The inverse effects of miR-610 down-regulation in MV3 cells: (B) proliferation promotion, (C) cycle progression promotion and (D) apoptosis inhibition. n = six independent experiments. *P<0.05.

Melanoma cell lines contained different endogenous miR-610 levels, were transduced in the cause of detecting the biological effects of miR-610 in melanoma progression. The qRT-PCR analysis showing in Figure 2A, we confirmed that miR-610 effectively upregulated miR-610 in A375 (P < 0.05) or downregulated miR-610 in MV3 cells (P < 0.05). Then BrdU incorporation assays and flow cytometric analysis were carried out, and the result had been shown in Figure 2B. Analyzing the data, it could be clearly found the miR-610 overexpression had a expressively effect on proliferation (P < 0.05) and cycle progression (P < 0.05) inhibition of A375 cells. And increased percentage of apoptotic cells was obviously obtained (P < 0.05) in Figure 2D. Oppositely, knockdown of miR-610 could clearly promote the proliferation (P < 0.05) and cycle progression (P < 0.05) of MV3 cells in Figure 2B and 2C. However, the attenuated percentage of apoptotic cells was markedly surveyed in Figure 2D (P < 0.05). In short, all the data indicated that miR-610 has critical effects on proliferation, cycle and apoptosis of melanoma cells.

### miR-610 suppresses the growth of melanoma *in vivo*

**Figure 3:**
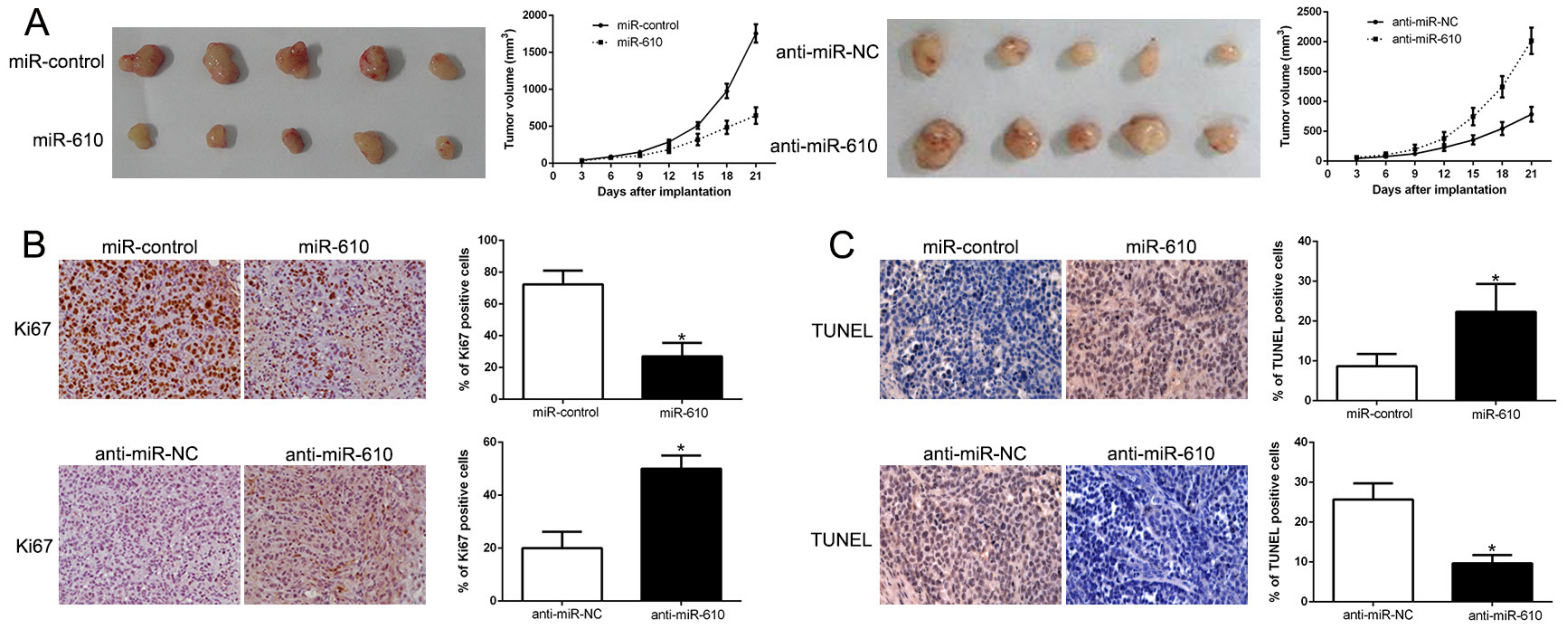
MiR-610 inhibits tumor growth and promotes apoptosis *in vivo* **(A)** Characteristic pictures of melanoma xenografts from both A375-miR-610 (left panel) and MV3-anti-miR-610 cells (right panel) (n=5). Tumor nodules were assayed and quantitatively analyzed by immunohistochemical staining for Ki-67 **(B)** and TUNEL **(C)**. Characteristic immunostaining and TUNEL assays manifested that the amount of Ki-67 positive cells was memorably reduced because of miR-610 overexpression while the amount of apoptotic cells was improved. However, the results were reversed in positive and negative control when miR-610 was knocked. *P<0.05.

In the further study, the subcutaneous tumor model and the tumor growth curves were established to recognize the functional significance on melanoma cells. And the experimental result disclosed that the growth of melanoma cells in mice was facilitated when miR-610 was knocked out (P < 0.05, Figure 3A) though unduly expression of miR-610 could suppress the tumor growth. Next, immunohistochemistry for Ki67 and TUNEL assays was performed in the xenografted tissues. When the miR-610 expressed overly, the number of cells staining positive for Ki67 was retarded and number of apoptotic cells for TUNEL positive was improved (P < 0.05) in Figure 3B and 3C. However, knocking miR-610 out increased the proliferation cells number and lessened the apoptotic cells number (P < 0.05). Taken all together, our data displayed that miR-610 could inhibit tumor progression of melanoma *in vivo*.

### miR-610 effects on cell cycle and apoptosis associated molecular

**Figure 4:**
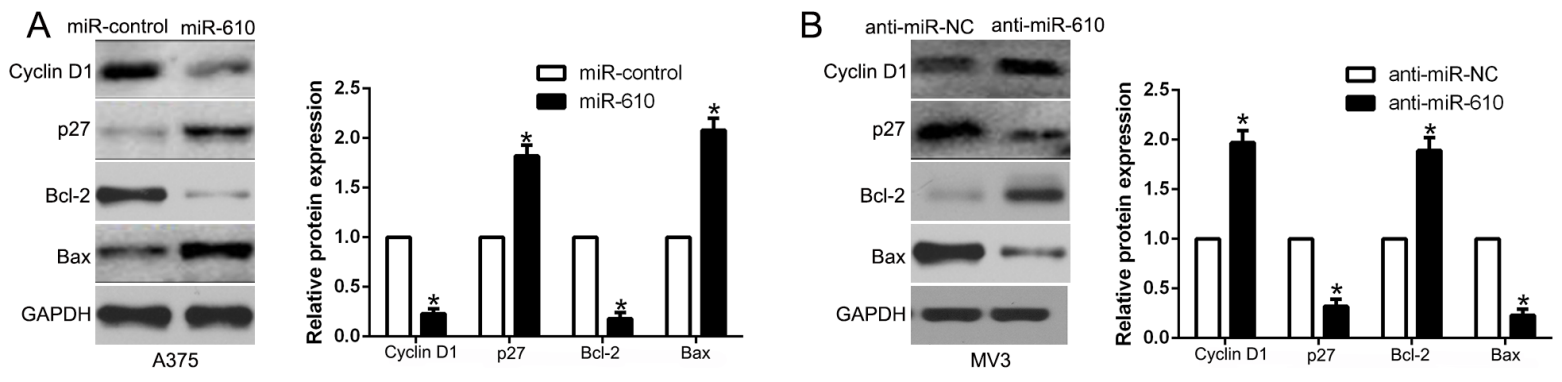
Western blot analysis of cycle regulator Cyclin D1 and p27, apoptosis-related protein Bcl2/Bax expression in the up-regulation **(A)** and down-regulation **(B)** of miR-610. n = six independent experiments. *P<0.05.

We performed western blot to probe the underlying mechanisms of miR-610 associated with melanoma cells. And the result was shown in Figure 3. It confirmed that up-regulated miR-610 markedly inhibited cycle-related protein, Cyclin D1 and apoptosis-inhibition protein Bcl-2. Whereas, cycle inhibitor p27 and pro-apoptosis protein Bax were prefered (P < 0.05, Figure 4A). Comparatively, miR-610 knockdown led to the enhancement of Cyclin D1, Bcl-2 and reduction of p27 and Bax (P < 0.05, Figure 4B).

### The role of LRP6 of miR-610 in melanoma cells

**Figure 5:**
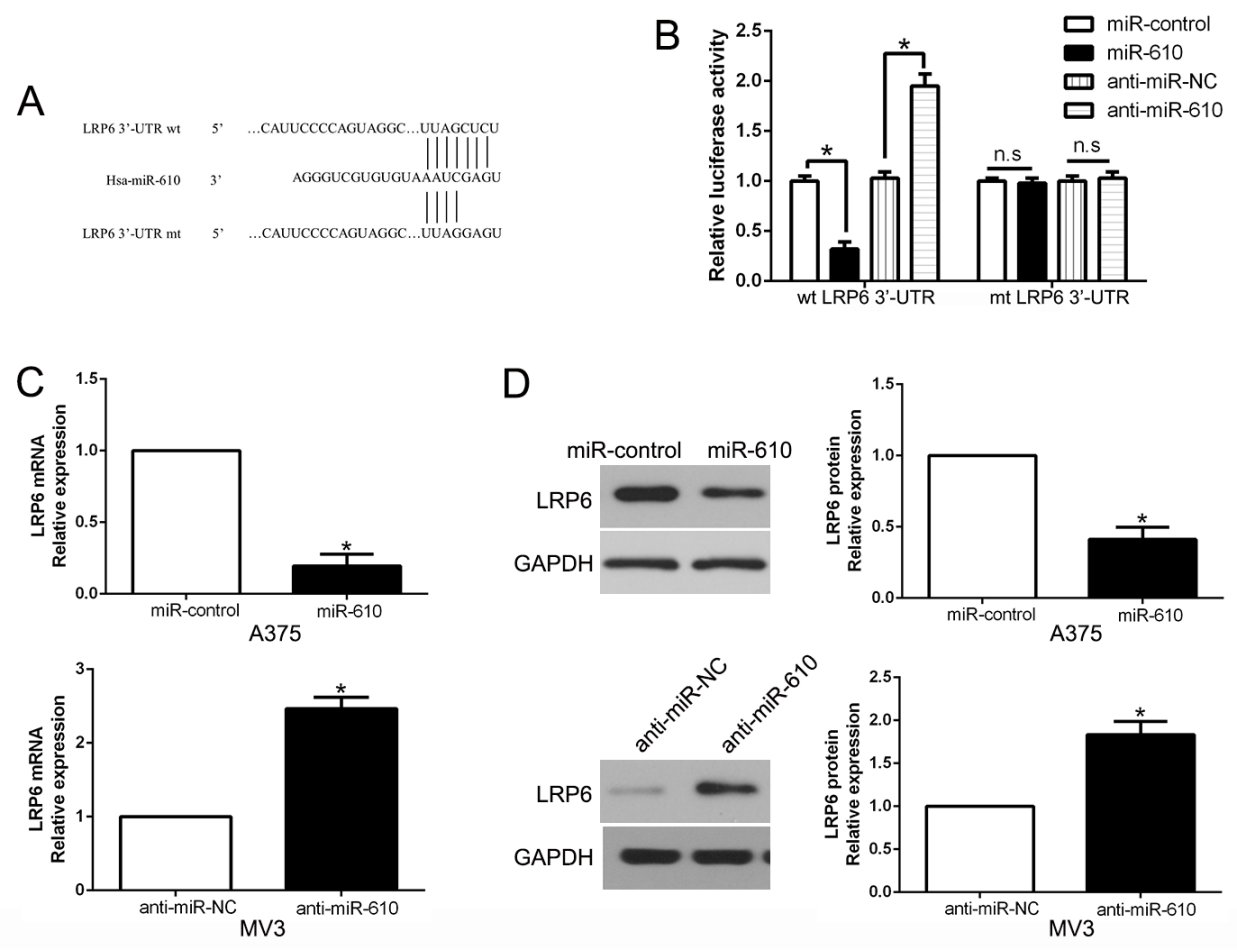
The role of LRP6 as a forthright target of miR-610 in melanoma **(A)** miR-610 and its putative binding sequence in the 3’-UTR of LRP6. **(B)** The luciferase activity of LRP6 was evidently inhibited by miR-610. **(C)** qRT-PCR analysis of LRP6 mRNA expression in A375 and MV3 cells. **(D)** The effect of miR-610 overexpression and knockdown on the level of LRP6 protein in A375 cells and MV3 cells, respectively. *P<0.05.

Candidate target was searched in bioinformatics database (TargetScan and Microrna) for the sake of determining the direct target of miR-610. And LRP6 was discovered to be a possible target (Figure 5A). Then luciferase reporter assay was performed for the aim of confirming the postulation that miR-610 could bind the 3’-UTR to LRP6. The experimental data clearly exhibited that overexpression of miR-610 restrained, while the luciferase activity of wild-type (wt) LRP6 3’-UTR was accelerated by miR-610 knockdown, but not mutant (mt) LRP6 3’-UTR (P < 0.05) in Figure 5B. Besides, the mRNA and protein expression of LRP6 in A375 cells were significantly restrained when miR-610 expression was excessive (P<0.05) in Figure 5C and 5D, respectively. By contrast, the inhibition of miR-610 in MV3 cells could signally augment the expression of LRP6 mRNA and protein (P<0.05).

### miR-610 expression was conversely correlated with LRP6 in melanoma tissues

**Figure 6:**
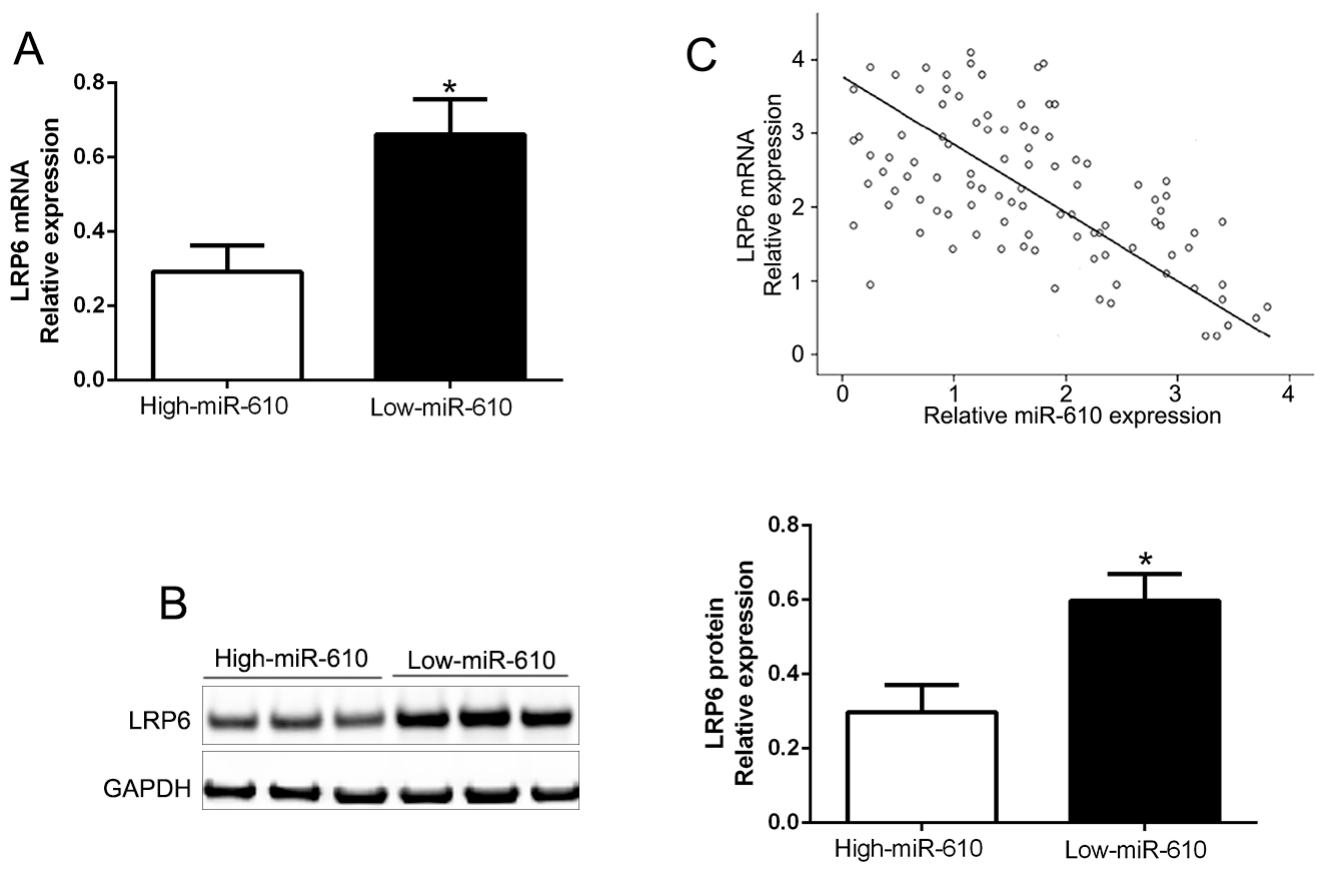
Inverse relevance between miR-610 and LRP6 expression in melanoma In miR-610 high-expressed tumors (n=52), LRP6 mRNA expression **(A)** and LRP6 protein **(B)** was expressively lower than corresponding expression in miR-610 low-expressed tumors (n=53). **(C)** Inverse relevance between mRNA levels of LRP6 and miR-610 was evidently surveyed in melanoma tissues. *P<0.05.

The mRNA and protein expression in varied miR-610 groups were measured to further check the association between miR-610 and LRP6 in tissues, and the results were shown in Figure 6. Comparing with low miR-610 group, LRP6 mRNA and protein levels were distinct lower in high miR-610 group (P<0.05, Figure 6A and 6B). Additionally, we exposed that the mRNA level of LRP6 was conversely related to miR-610 expression (R^2^=0.6458, P<0.0001, Figure 6C). In addition, we performed western blot and found that LRP6 was increased in a panel of melanoma cell lines compared HPM (P<0.05, Supplementary Figure 1A). Moreover, the data showed the expression of LRP6 in melanoma tissues was significantly higher than that in non-tumor tissues (P<0.05, Supplementary Figure 1B). Throughout the data, a conclusion could be obtained that LRP6 was a direct downstream target of miR-610 in melanoma.

### Recovery of LRP6 expression partially inversed the antitumor effects of miR-610 on melanoma cells

**Figure 7:**
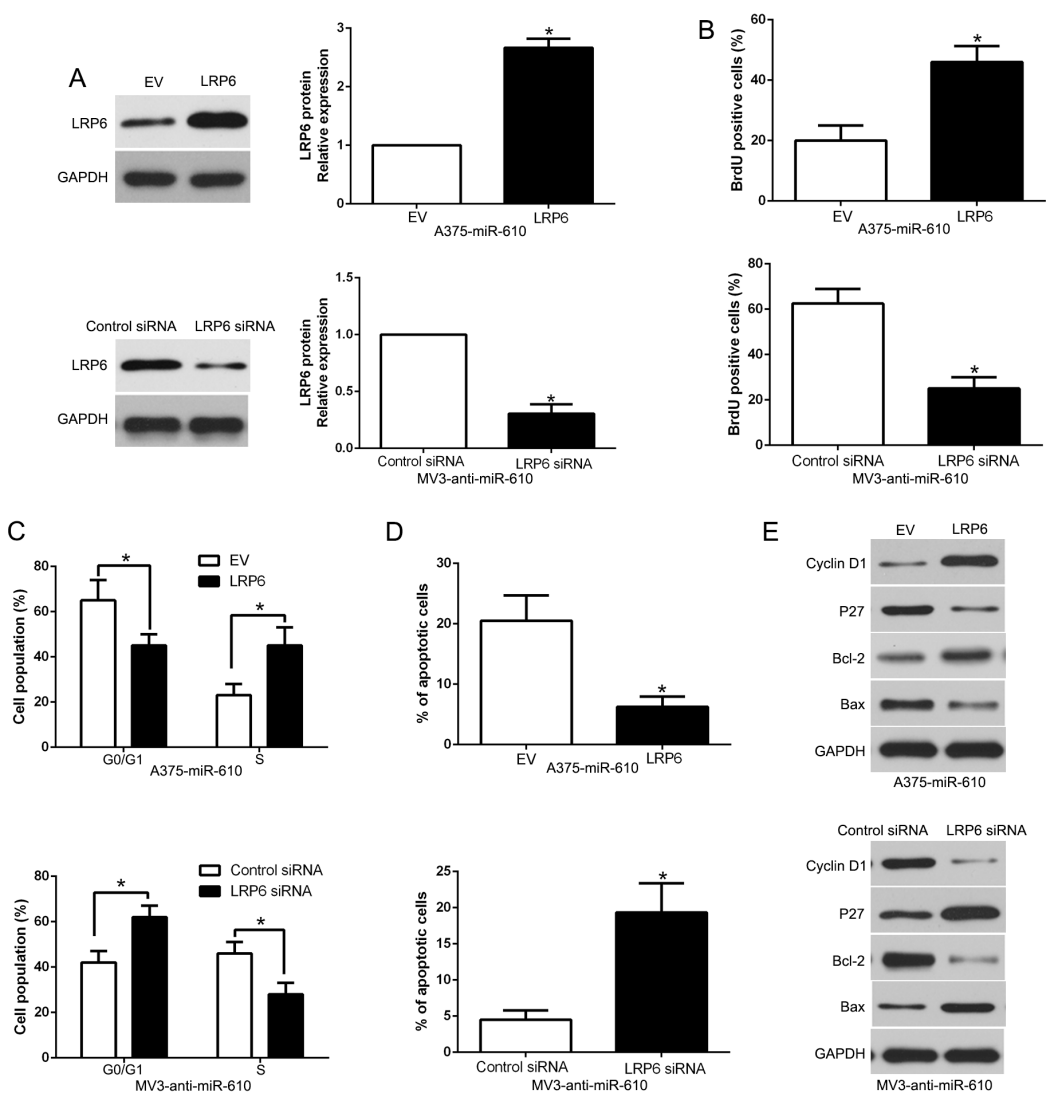
Alterations of LRP6 partially abrogate miR-610-mediated proliferation, cycle progression and apoptosis of melanoma cells **(A)** Western blot was carried out for analyzing the levels of LRP6 in miR-610-overexpressing A375 cells and miR-610-suppressive MV3 cells. The biological effects of LRP6 knockdown or overexpression form miR-610: **(B)** proliferation, **(C)** cycle progression and **(D)** apoptosis. **(E)** Effects of LRP6 restoration on cell cycle and apoptosis-related regulators. n = 3 (independent experiments were implemented). ^*^P < 0.05.

To detect whether LRP6 interceded the effects of miR-610, we restored LRP6 expression by making plasmid express overly in miR-610-overexpressed A375 cells (P<0.05) in Figure 7A. Our data showed that restituted LRP6 could partially abrogate the effect of miR-610, resulted in enhancement of proliferation, cycle progression and apoptosis inhibition in miR-610-overexpressed A375 cells (P<0.05, Figure 7B-7E, Supplementary Figure 2). Equally, the biological effects of anti-miR-610 were partially abrogated by silencing of LRP6 using a specific RNA interference in miR-610-suppressive MV3 cells (P<0.05). These results demonstrated that LRP6 is a downstream adjuster of miR-610 in melanoma.

## DISCUSSION

There has research evidence that the development and progression of melanoma is concerned with aberrant miRNAs through the play of possible prognostic biomarkers and effective therapeutic targets [20]. In present study, our study verified that miR-610 expression in melanoma cell lines and tissues was decreased. It has been reported that the miR-610 repressed CCND2 and AKT3 leading to proliferation inhibition on human glioblastoma cells [21]. The literature of Yan et al proved that the downregulation of miR-610 in glioma cells is clear, and it can inhibit proliferation and motility by forthrightly targeting MDM2 [22]. Moreover, the truth that miR-610 was underexpressed in buccal mucosa of oral submucous fibrosis was discovered by Zhang et al using microarray assay [23]. The results in previous studies reveal a tumor-suppressive duty of miR-610. In this research, our results indicate that miR-610 functions as a negative regulator in melanoma.

The explicit responsibility of miR-610 in melanoma remains unknown though the play of miR-610 as a tumor suppressor was reported by many research. In present study, clinical analysis exposed that the reducing of miR-610 was signally associated with malignant clinicopathological features of melanoma patients. Moreover, terrible effect of low miR-610 group for 5-year survival of melanoma patients had be found. Basis on the explored data, our results indicated that miR-610 is decisive for prognosis outcome of melanoma patients. Functionally, according to the gain- and loss-function experiment results, it could be found that miR-610 possessed the function of inhibiting proliferation, cycle progression and promoted apoptosis, at least partially by targeting LRP6 *in vitro* and *vivo*. Furthermore, miR-610 was inversely associated with LRP6 expression, which was overexpressed in melanoma tissues. The accumulation of LRP6 in melanoma cells was also negatively regulated by miR-610. In general, these results uncovered that direct restraint of LRP6 make miR-610 become a tumor suppressor acting on proliferation, cycle progression and apoptosis inhibition of melanoma.

LRP6 is one member of low-density lipoprotein receptor family, and it also belongs to Wnt co-receptors [24]. When the translocation of β-catenin is improved, the transcription of Wnt/β-catenin target genes could be activate by LRP6 [25]. Moreover, it can be found that LRP6 expresses excessively in different cancers, including melanoma [26-29]. Abnormal activation of Wnt/β-catenin signaling pathway has considerable effect on tumor progression [17, 30, 31]. Notably, our luciferase assay confirmed that miR-610 could binds to the 3’-UTR of LRP6. And we found LRP6 pathway had been used by miR-610 to regulate cell cycle regulator Cyclin D1, p27 and apoptosis-associated factors Bcl-2, Bax. In brief, all results certified the remarkable responsibility of miR-610 in melanoma.

Conclusively, miR-610 was down-regulated in melanoma cell lines and tissues in the present study. And we manifested insufficient miR-610 expression was concerned in malignant clinicopathological features. Furthermore, directly targeting LRP6 was an useful pathway for miR-610 to inhibit cell proliferation, cell cycle progression and promoted apoptosis. Our results confirmed that miR-610 exist in melanoma as a potential tumor suppressor biomarker. Taken together, the unusual regulation of miR-610 may has an momentous function in tumor growth process. Hence, miR-610 may become a latent prognostic factor and latent therapeutic target for melanoma.

## MATERIALS AND METHODS

### Clinical tissues and cell culture

Department of Dermatology (Cangzhou Central Hospital) offered the melanoma tissues and matched adjacent non-tumor during January 2006 to December 2010. None of interventional was performed before surgery. All patients knew this research and signature on the informed consent. Ethical Committee of Cangzhou Central Hospital approved the research.

The melanoma cell lines (SK-MEL-1, A375, SK-MEL-28, MV3 and B16-F1) and human primary melanocytes (HPM) were gained from the American Type Culture Collection (Manassas, VA, USA) and PromoCell (Beijing, China), respectively. The RPMI-1640 (Gibco, Carlsbad, CA, USA), which contained 10% of FBS (Invitrogen, Carlsbad, CA) and 1% of penicillin-streptomycin (Sigma, St. Louis, MO, USA) was used for culturing the cells under the condition of 37°C with 5% CO_2_.

### Quantitative reverse transcriptase polymerase chain reaction (qRT-PCR)

Total RNA samples were isolated from tissue samples or cells utilizing TRIzol reagent (Thermofisher Scientific, Inc., Carlsbad, CA, USA). To obtain cDNA, reverse transcription was conducted through All-in-One miRNA qRT-PCR Detection kit (GeneCopoeia, Rockville, MD, USA). The PCR experiment was accomplished using the same apparatus with a Bio-Rad iCycler single-color real-time detection system (Bio-Rad Laboratories, Inc., Hercules, CA, USA). Utilizing delta-delta Ct method with U6 or GAPDH as an internal control assessed the gene expression levels. Hsa-miR-610 primer (HmiRQP0714), snRNA U6 qPCR Primer (HmiRQP9001), LRP6 (HQP010877) and GAPDH (HQP006940) were obtained from Genecopoeia (Guangzhou, China).

### Lentivirus transduction and oligonucleotide transfection

The lentiviral particles for miR-610 overexpression and inhibition constructs were parceled and bought from GeneChem (Shanghai, China). Recombinant lentivirus was adopted for infecting A375 and MV3 cells transducing units plus 5 μg /ml Polybrene (Sigma, Natick, MA, USA). Then cells were collected 48 h after transduction.

LRP6 vectors, including LRP6 expression vector, empty vector, LRP6 siRNA and control, were acquired from Genepharm Co. Ltd (Shanghai, China). The LRP6 plasmid, LRP6 siRNA and a scramble siRNA were gained from Sangon Biotech Co., Ltd. (Shanghai, China). Using six-well plate seeded cells with a concentration of 2×10^6^ per well. Employing Lipofectamine 2000 Reagent (Invitrogen Life Technologies) transfected the treated cells with 100 nm above vectors.

### Western blot analysis

RIPA buffer was used for lysing the tissues or cells. Subsequently, BCA Protein Assay Kit bought from Tiangen (Beijing, China) was engaged to estimate the concentrations. 30 μg of protein was treated with 10% of SDS-PAGE gel, then transferred onto PVDF membranes (Millipore, Billerica, MA, USA). 5% non-fat milk was the used to block the membranes in TBST for 2h at r.t. Then specific primary antibodies (1:1000, Cell Signaling Technology, Inc.) was adopted to hatch the above mixture overnight at 4°C. HRP-conjugated secondary antibody (ZSGB-BIO, China) was then carried out to incubate the membranes at room temperature for 2h. To detect experimental data, enhanced chemiluminescence kit (Amersham, Little Chalfont, UK) was operated. The quantification of protein bands intensity was measured by Quantity One software 4.5.0 basic (Bio-Rad).

### Detection of proliferation, cell cycle and apoptosis

Cells were seeded in 24-well plate for assaying the proliferation, hatched with BrdU for 1.5 h. Then staining process was conducted using anti-BrdU antibody (Sigma, St. Louis, MO, USA). Afterwards, using fluorescence-activated cell sorting (FACS) Calibur with Cell Quest software (Becton-Dickinson, San Jose, CA, USA) executed the flow cytometry. Cells were seeded in 6-well plates at 2x10^5^/well and transfected for 48 h for sake of assaying the cycle. Cells were then stained with 50 μg/ml of PI (Keygen, Nanjing, China) after fixing in 70% of alcohol at 4°C for 24 h. Then apoptosis levels were appraised by an Annexin-V-Fluos Staining kit (Roche).

### Immunohistochemical staining (IHC)

Briefly, 4 μm sections were deparaffinized in xylene, rehydrated through graded ethanol, followed by blocking of endogenous peroxidase activity in 4% hydrogen peroxide for 10 min at room temperature. The corresponding antibody Ki67 (1:300) was applied as the primary antibody by a streptavidin peroxidase-conjugated (SP-IHC) method. The staining results were semi-quantitatively evaluated by the multiply of staining intensity and the percentage of positive staining cells.

### Luciferase reporter assay

The 3’-UTR sequence of LRP6, together with a corresponding mutated sequence, were synthesized. They were then embedded into the pmiR-GLO dual-luciferase miRNA target expression vector (Promega, Madison, WI, USA), named wt-LRP6 3’-UTR and mt-LRP6 3’-UTR, respectively. After plating into 24-well plate, miR-610 inhibitor or negative control was accepted for transfecting A375 cells. Then using Lipofectamine 2000 reagent (Invitrogen, USA) co-transfected the cells with wild-type or mutant 3’-UTR of LRP6 vector for 48h. Subsequently, cells were gathered and measured using Dual-Luciferase Assay (System; Promega). To correct the efficiencies of transfection and gather, the Renilla luciferase expressed by pRL-TK was co-transfected as an internal control.

### *In vivo* experiments

Four-to-six-week-old female BALB/c mice were adopted to establish the nude mouse xenograft model for further research. A375 (5x10^6^) cells, which were transduced with miR-610 or miR-control vectors or MV3 cells with anti-miR-610, were mingled in 150 μl of Matrigel. Then the flank of nude mice was handled by subcutaneous injection with mixture. The equation for calculate tumor volume was the follow: tumor volume = length × width × width/2. The Institutional Animal Care and Use Committee of Cangzhou Central Hospital approved the animal protocols.

### Statistical analysis

Independent experiments were performed at least three times. The software of SPSS 16.0 (Inc, Chicago, IL, USA) and Graphpad Prism 6.0 (CA, USA) were applied to analyze the experimental data. Besides, the statistical significance was also evaluated. All data were displayed as the mean ± SD. Differences were also defined as P<0.05.

## SUPPLEMENTARY MATERIALS FIGURES


