# Supplementary material for: MicroRNA-610 inhibits tumor growth of melanoma by targeting LRP6
Source: Oncotarget. 2017 Oct 26;8(57):97361–70. doi: 10.18632/oncotarget.22125 (PMC5722568; doi:10.18632/oncotarget.22125)
Supplement: Supplementary file 1 [file oncotarget-08-97361-s001.pdf]

# MicroRNA-610 inhibits tumor growth of melanoma by targeting LRP6

## SUPPLEMENTARY MATERIALS

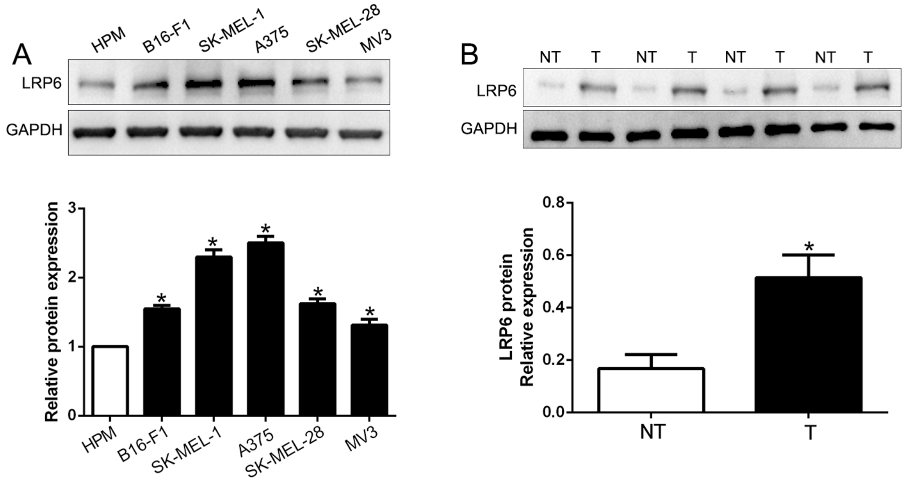


**Supplementary Figure 1:** The expression of LRP6 in melanoma cell lines **(A)** and tissues **(B)**.

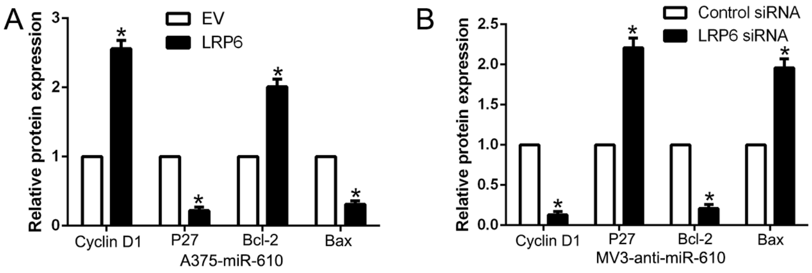


**Supplementary Figure 2:** The densitometric analysis of Figure 7E of LRP6 alteration changed the cell cycle and apoptosis-related regulators after LRP6 overexpression **(A)** and knockdown **(B)**.
